# Geriatricians’ role in the management of aortic stenosis in frail older patients: a decade later

**DOI:** 10.1007/s41999-024-01015-9

**Published:** 2024-07-22

**Authors:** Andrea Ungar, Giulia Rivasi, Giuseppe Dario Testa, Anne Sophie Boureau, Francesco Mattace-Raso, Manuel Martínez-Sellés, Mario Bo, Mirko Petrovic, Nikos Werner, Athanase Benetos, on behalf of the EuGMS Special Interest Group on Cardiovascular Medicine

**Affiliations:** 1 Geriatrics and Intensive Care Unit, University of Florence and Azienda Ospedaliero Universitaria Careggi, Florence, Italy; 2 Nantes Université, CHU Nantes, Pole de Gérontologie Clinique, 44000 Nantes, France; 3 Division of Geriatrics, Department of Internal Medicine, Erasmus MC University Medical Center, Rotterdam, The Netherlands; 4 Cardiology Department, Hospital General Universitario Gregorio Marañón, Instituto de Investigación Sanitaria Gregorio Marañón, CIBERCV, Universidad Europea, Universidad Complutense, Madrid, Spain; 5 Section of Geriatric, Department of Medical Sciences, University of Turin, Città della Salute e della Scienza, Molinette, Turin, Italy; 6 Section of Geriatrics, Department of Internal Medicine and Paediatrics, Ghent University, Ghent, Belgium; 7 Heart Center Trier, Department of Internal Medicine III, Krankenhaus der Barmherzigen Brüder Trier, Trier, Germany; 8 Geriatric Department and Federation Hospital-University On Cardiovascular Aging (FHU-CARTAGE), University Hospital of Nancy, Université de Lorraine, Vandoeuvre-Lès-Nancy, France

**Keywords:** Transcatheter aortic valve implantation, Heart team, Frailty, Comprehensive geriatric assessment, Aortic valve replacement

## Abstract

**Introduction:**

Increasing evidence supports the implementation of geriatric assessment in the workup of older patients with aortic stenosis (AS). In 2012, an online European survey revealed that geriatricians were rarely involved in the assessment of candidates for transcatheter aortic valve implantation (TAVI). After a “call to action” for early involvement of geriatricians in AS evaluation, the survey was repeated in 2022. Our aim was to investigate whether geriatricians’ role changed in the last decade.

**Methods:**

Online survey conducted between December 16th, 2021, and December 15th, 2022. All members of the European Geriatric Medicine Society were invited to participate. The survey included 26 questions regarding geriatricians’ experience with AS and TAVI.

**Results:**

Among 193 respondents (79.8% geriatricians), 73 (38%) reported to be involved in AS evaluation at least once a week. During 2 years prior to the survey, 43 (22.3%) had referred > 50% of their patients with severe AS for TAVI. Age influenced TAVI referral in a considerable proportion of respondents (36.8%). TAVI candidates were mainly referred to specialised cardiac centres with multidisciplinary teams (91.8%), including (47.2%) or not including (44.6%) a geriatrician. A total of 38.9% of respondents reported to be part of a multidisciplinary heart team. Geriatricians were less frequently involved (37%) than cardiologists (89.6%) and surgeons (53.4%) in pre-procedural TAVI management. Cardiologists were more frequently involved (85.5%) than geriatricians (33.7%) and surgeons (26.9%) in post-procedural management.

**Conclusions:**

Geriatricians’ involvement in AS management and multidisciplinary heart teams remains scarce. More efforts should be devoted to implement geriatricians’ role in AS decision-making.

**Supplementary Information:**

The online version contains supplementary material available at 10.1007/s41999-024-01015-9.

## Key summary points

**Aim:**

The objective of this survey was to investigate geriatricians’ role in the management of older patients with aortic stenosis, over the last decade.

**Findings:**

Our results indicate that aortic stenosis is a common disease at older age and frequently coexists with geriatric syndromes. Nevertheless, geriatricians’ role in multidisciplinary assessment of aortic stenosis is scarce, similarly to what reported in a previous survey conducted a decade ago.

**Message:**

More efforts should be devoted to implement geriatricians’ involvement in aortic stenosis management and multidisciplinary heart teams.

**Supplementary Information:**

The online version contains supplementary material available at 10.1007/s41999-024-01015-9.

## Introduction

Aortic valve stenosis (AS) is highly prevalent in old age [1]. While the prognosis is relatively benign in asymptomatic patients, the presence of symptoms is associated with an increased risk of mortality, with a median survival of 1–3 years [2]. In recent years, transcatheter aortic valve implantation (TAVI) has emerged in randomized trials involving high [3], intermediate [4], and low-risk patients [5]. TAVI is now the treatment of choice for patients older than 75 with symptomatic severe AS [6]. However, a considerable proportion of these patients show no improvement in symptoms, quality of life, and functional autonomy [7–9], revealing that some flaws exist in treatment decision-making processes and risk stratification tools, which are mainly based on chronological age, comorbidities and cardiological but not geriatric parameters. These observations underscore the need of optimizing patients’ risk stratification and referral to different treatment strategies. The goal is to more effectively recognize potential therapeutic benefits, and, conversely, identify procedures that, although successful, may not significantly improve health outcomes.

Over recent years, a growing body of evidence has emphasized the prognostic relevance of comprehensive geriatric assessment (CGA), showing a greater predictive value for adverse outcomes than traditional surgical risk scores [10, 11]. In particular, functional status, physical performance, and frailty have been identified as the strongest predictors of functional decline, deterioration of health-related quality of life, and mortality after TAVI [7, 12–16]. The abovementioned evidence suggests the implementation of CGA in the workup of older patients with AS, to help distinguish older individuals who may benefit from intervention from those who will gain little benefit or may even be harmed. Indeed, geriatric parameters such as functional status and physical performance were found to significantly influence treatment decision-making, independently of cardiac components, when CGA was performed during symptomatic AS pre-operative evaluation [17, 18]. Moreover, CGA might prompt tailored interventions, e.g., nutritional support or medical therapy optimization, that significantly impact patients’ health status, beyond the outcomes of the procedure.

These data are consistent with recent cardiovascular literature showing a substantial prognostic impact of frailty in older patients with cardiovascular diseases [19–21]. In this context, a recent position statement by the EuGMS Special Interest Group (SIG) on Cardiovascular Medicine has advocated the implementation of a geriatric approach in the management of cardiovascular diseases in older adults. Indeed, a geriatric approach may allow for providing holistic patient-centred care, customized to the individual’s functional status and level of frailty, and focused on functional autonomy and quality of life [22].

In 2012, the EuGMS SIG on Cardiovascular Medicine invited geriatricians to participate in an online survey regarding their experience with AS, with reference to their involvement in treatment decision-making and assessment of TAVI candidates (Supplementary Table 1) [23]. The survey showed that geriatricians rarely played an active role in AS management, being involved only in 35% of cases during pre-procedural assessment and in 50% of cases during post-procedural assessment. Moreover, among geriatricians participating in the survey, only one-fifth were part of a multidisciplinary heart team [23]. These results suggested the considerable potential for the implementation of geriatric assessment and led to a ‘‘call to action’’ of the EuGMS claiming for the involvement of geriatricians in early phases of the AS workout. In particular, the EUGMS recommended the integration of geriatricians into multidisciplinary heart teams. Their active involvement in decision-making is advised not only concerning treatment strategies but also in matters related to long-term care and rehabilitation. However, it is unclear whether geriatricians’ role has been implemented in the following years.

The EuGMS SIG on Cardiovascular Medicine thus decided to repeat the same survey in 2022, with the final purpose to investigate whether geriatricians’ involvement in AS management has been changed in the last decade. The present paper is aimed to illustrate the survey results and discuss how geriatricians’ involvement has evolved over the last decade.

## Methods

The survey was conducted between December 16th, 2021, and December 15th 2022, through online English questionnaire formatted using Google Forms. All members of the European Geriatric Medicine Society (EuGMS) were invited to participate by e-mail, with a reminder sent every 2 months. The list of EuGMS members is regularly updated by the society secretariat and the invitation to complete the survey was sent to any new member registered during the study period. Participation in the survey was voluntary and confidential. The survey consisted of 26 questions investigating demographics and professional background (questions 1–6), experience with diagnosing/treating AS (questions 7–12), and experience with TAVI (questions 13–26). Compared to the previous 2012 version, two additional questions were included regarding professionals involved in the assessment of TAVI candidates (question 25) and the possible influence of age on the decision to refer patients for TAVI (question 26). The survey mainly included multiple-choice questions, with some semi-open questions (i.e., others, please specify). The full questionnaire is detailed in Supplementary Table 2.

Data are presented as means with standard deviations (SD) for continuous variables and as absolute frequencies with percentages (*n*, %) for categorical variables. All statistical analyses were performed using SPSS software version 26 (SPSS, Inc., Chicago, IL).

## Results

### Demographic and professional background

**Fig. 1:**
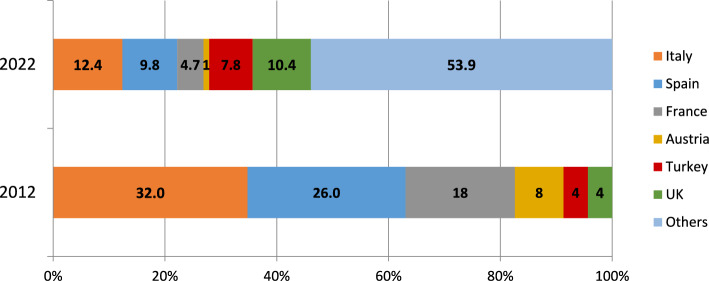
Respondents’ countries of origin in 2012 and 2022 surveys. *UK* United Kingdom

**Fig. 2:**
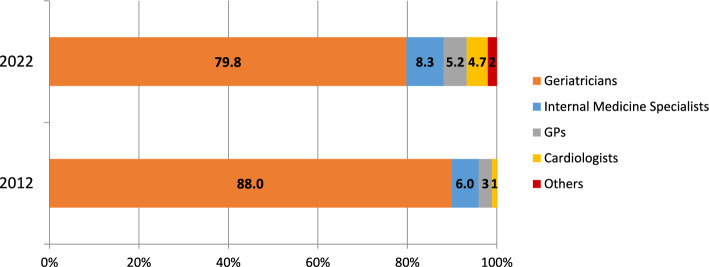
Respondents’ medical specialty in 2012 and 2022 surveys. *GPs* general practitioners

A total of 193 physicians participated in the survey (48.7% women). As depicted in Fig. 1, the largest proportion of respondents were based in Italy (12.4%), followed by the United Kingdom (10.4%) and Spain (9.8%). Most participants (62.7%) were aged 35–54 years, while 16.6% were aged 25–35 years, and approximately one-fifth (20.7%) were 55 or older. Respondents mainly included geriatricians (79.8%), followed by internal medicine specialists (8.3%) (Fig. 2). Most participants reported over 10 year experience in their speciality (10–20 year experience: 36.3%; over 20 year experience: 26.9%. Approximately 49% of them spent > 50% of their working activity in the acute care setting, whereas 21% and 11% of participants dedicated > 50% of their working activity to rehabilitation and long-term care, respectively.

### Experience in the treatment of aortic stenosis

**Fig. 3:**
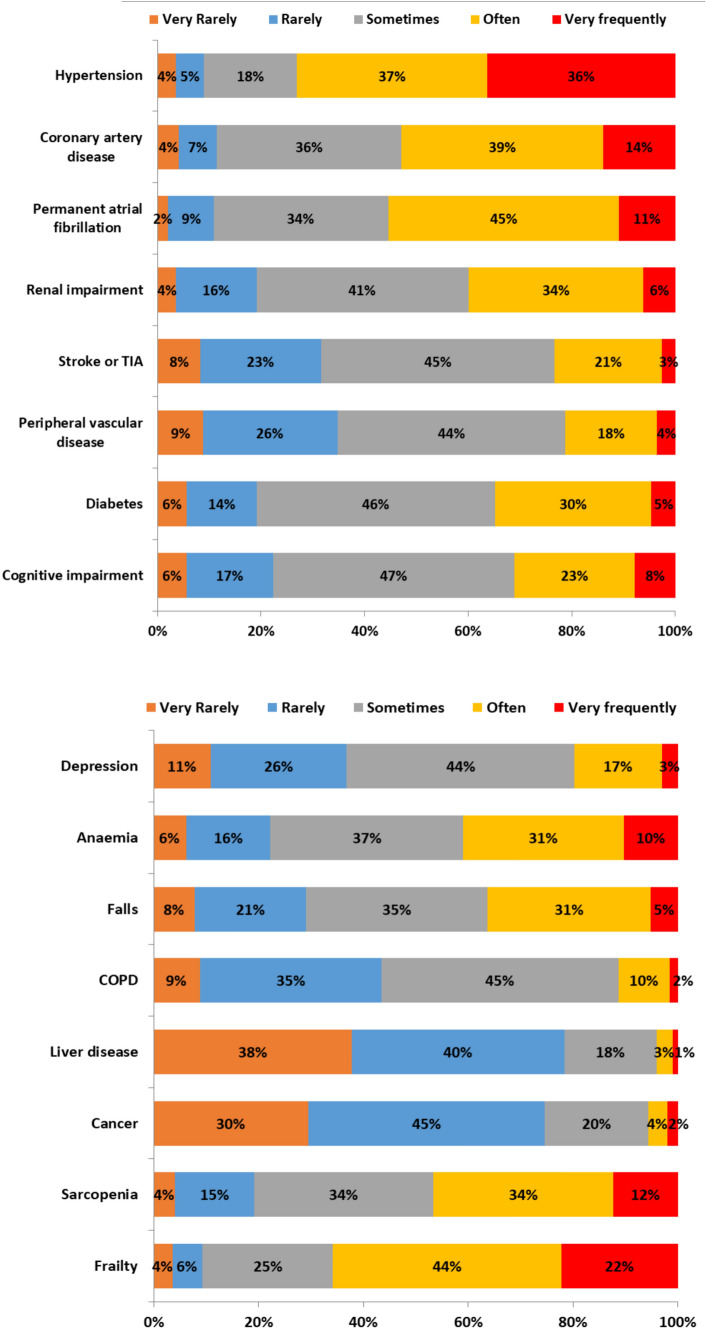
Frequency of co-morbidities and geriatric syndromes in patients with aortic stenosis. *TIA* transient ischemic attack; *COPD* chronic obstructive pulmonary disease

When experience with AS was investigated, 38% of respondents reported to be involved at least once a week in the management of older patients with AS. Regarding disease severity, 12% of participants reported that AS was severe in more than half of patients they had evaluated in 3 months prior to the survey. The most frequent symptoms associated with AS were dyspnoea (86%), fatigue (78.2%), and syncope (68%), while falls during effort (49%), angina (47%), and delirium (23.8%) were less frequently reported. Comorbidities most commonly associated with AS are detailed in Fig. 3. Sarcopenia and frailty (not investigated in 2012) were found to be prevalent or highly prevalent conditions in 46% and 66% of individuals with AS, respectively.

**Fig. 4:**
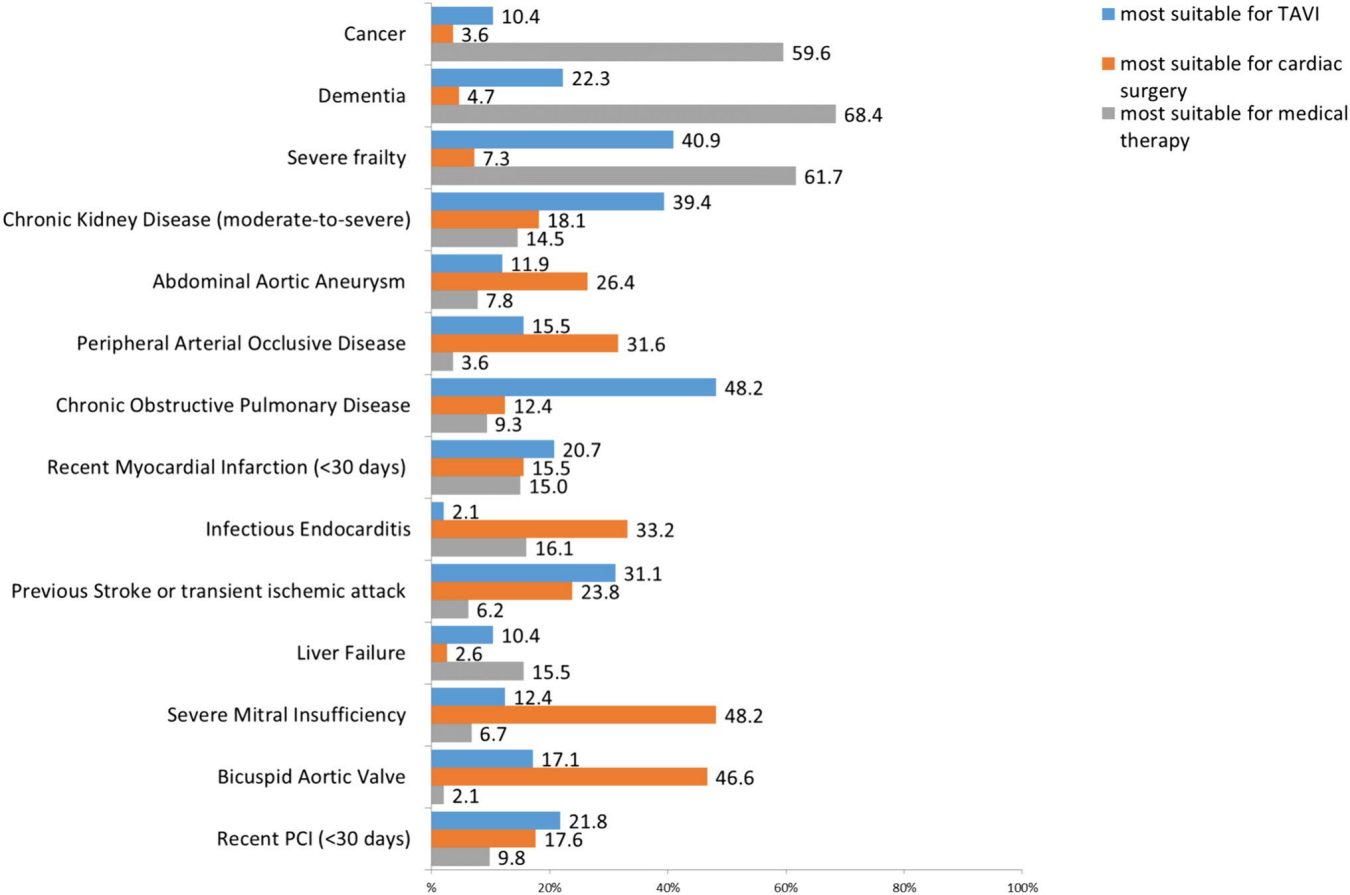
Most suitable treatment options for different comorbidities and geriatric syndromes (percentage). *CKD* chronic kidney disease; *PAD* peripherally artery disease; *MI* myocardial infarction; *TIA* transient ischemic attack; *COPD* chronic obstructive pulmonary disease; *PCI* Percutaneous Coronary Intervention

When AS treatment history was investigated, 55% of participants reported that over half of their patients had not previously received any specific therapy (i.e., surgery or other procedures). The 4.1% and 11.4% of respondents reported that most of their patients had already received surgery and TAVI, respectively. A list of conditions potentially influencing referral to surgical treatment vs TAVI was also investigated (Fig. 4). Chronic obstructive pulmonary disease, moderate to severe chronic kidney disease, and severe frailty were indicated as the conditions making patients more suitable for TAVI, while cancer, dementia, and severe frailty were identified as the predominant indications for medical therapy. A considerable proportion of participants (36.8%) reported that age significantly influenced their decision to refer patients for TAVI.

### Involvement in TAVI management

The 96.4% of respondents reported they would know where to refer a TAVI candidate. The most common option for referral was represented by specialized cardiac centres with multidisciplinary teams, including (47.2%) or not including (44.6%) a geriatrician. Respondents also referred TAVI candidates to general hospitals (5%) or cardiologists (3.1%).

During 2 years prior to the survey, 3.1% of respondents had referred more than half of their AS patients to cardiac surgery, while 22.3% and 31.6% had referred more than half of patients for TAVI and medical therapy, respectively. A total of 76 participants had referred no patients for TAVI in 2 years prior to the survey; the main reasons were limited life expectancy (*n* = 32), severe frailty (*n* = 23) and patients’ refusal (*n* = 15). The 37.8% and 32.5% of participants, respectively, reported functional improvement in > 50% of their TAVI patients within 3 months and beyond 3 months following the procedure. The 35.5% and 8.4% of participants reported NYHA improvement and cognitive improvement among most of their TAVI patients.

**Fig. 5:**
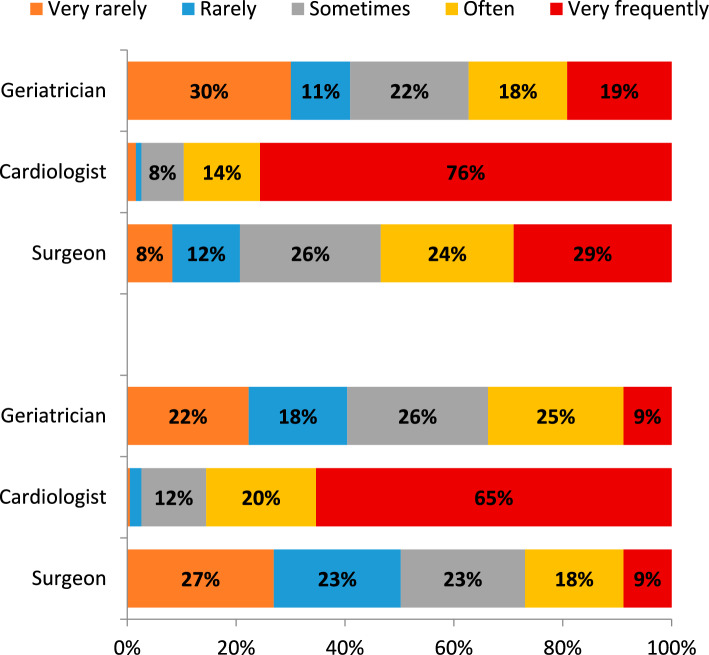
Involvement of different professionals in pre- (upper panel) and post-procedural (lower panel) TAVI management (often and very frequent involvement are indicated as “frequent” in the text)

Geriatricians were rarely involved in pre- and post-procedural TAVI management (Fig. 5). Notably, the 38.9% of respondents reported being part of a multidisciplinary team for management of TAVI candidates.

## Discussion

In the present EuGMS survey on AS and TAVI management, we observed that geriatricians’ role in multidisciplinary assessment remains scarce, although AS is common in the geriatric population. In addition, TAVI currently represents the treatment of choice for older patients with symptomatic severe disease.

Most of the respondents was geriatricians with over 10 year experience in their specialty, and most of them were frequently involved in the management of AS-patients, at least once a week for more than third of them, even though it was mainly patients with mild or moderate AS.

In this survey, prevalent clinical conditions reported by physicians were both cardiovascular comorbidities (hypertension, coronary artery disease, atrial fibrillation) and geriatric syndromes (sarcopenia, frailty, falls). This result is concordant with national database and larger studies which found high prevalence rates of cardiovascular diseases, renal insufficiency, diabetes, anaemia as well as frailty and sarcopenia [24]. Frailty and sarcopenia, not investigated in 2012, were encountered very frequently. In previous studies, the prevalence of sarcopenia in TAVI patients widely varied (between 21 and 70%) according to assessment methods and the included population [25]. Moreover, estimates of the prevalence of frailty among TAVI patients vary widely depending on the definition and tools used. Frailty increases the risk of morbidity, mortality, and poor quality of life across the spectrum of cardiovascular disease [15, 26]. However, frailty could be reversible after TAVI. Indeed, many of the markers of Fried frailty phenotype, such as slow gait speed or exhaustion, can simply represent symptomatic severe AS. When dyspnoea from severe AS limit physical function, TAVI has proved beneficial on patients’ functional capacity, potentially offering a chance to reverse frailty. To maximize the chance of reversibility—while avoiding futility—a comprehensive geriatric assessment is required, aiming to explore all factors which might influence physical function and frailty level, as well as TAVI outcomes [18].

Although frailty and sarcopenia are known to significantly impact older patients’ prognosis and TAVI outcomes, a standardized assessment has yet to be implemented in the workout of AS. As a result, these conditions are commonly overlooked in routine practice, particularly in non-geriatric settings. Future research should probably explore available instruments and their current application in AS pathways, with the final purpose to develop standardized strategies for assessment, treatment and monitoring of these geriatric syndromes in AS patients.

### Experience in the management of severe aortic stenosis

Since 2012, more patients have been referred for an interventional treatment of severe AS, also because of technical progress and developments periprocedural management. In European countries, TAVI accounted for an increasing proportion of all aortic valve procedures as age increases [27]. This result is explained by the good outcomes in randomized trials involving high [7], intermediate [8], and low risk patients [9] as well as the recent European guidelines on AS management [6] as treatment of choice for older patients with symptomatic severe AS. However, in the present survey, nearly one third of respondents reported to have referred more than half of their AS patients to medical therapy. This proportion is higher than in the European Heart Survey on Valvular Heart disease conducted in 2019 which found that only 30.6% of AS-patients did not underwent any kind of intervention [28]. A relevant proportion of respondents did not consider severe frailty a contraindication to interventional therapy of AS, including surgery. This might be at least partly related to the lack of a routine and standardised assessment of frailty by different professionals and the consequent lack of agreement on the definition of severe frailty in AS patients. By contrast, most participants advocated medical therapy only for cancer patients and those with dementia. According to international guidelines, TAVI can be considered unless life expectancy is less than 1 year. Our data suggest that some individuals with mild dementia or early stage cancer—who might benefit from interventional AS therapy—might instead be excluded from these treatment options despite their life expectancy can be supposed to be longer than 1 year.

More than one third of the respondent acknowledged that their choice was influenced also by patients’ age. Numerous studies have shown that TAVI in nonagenarians is a viable and safe treatment option [29, 30]. Therefore, oldest patients should not be denied this treatment option based on age alone. However, the geriatric approach requires that selection of candidates for TAVI is based on individualized estimates of procedural risk, potential for functional recovery and for improved quality of life as in all geriatric patient’ management regardless of age.

Another reason for older AS-patients remained treated with medical therapy is the difficulty to assess patient symptoms. Indeed, signs and symptoms of aortic stenosis may be difficult to recognize at old age, particularly in frailer patients, due to the reduced physical activity associated and concomitant conditions that might be responsible for similar symptoms. Moreover, reduced physical activity is frequently attributed to old age itself, thus potentially underestimating activity restriction related to AS Therefore, a detailed medical history and careful functional and clinical assessment with patients and their families are extremely important with a view to develop customized treatment strategies.

### Involvement in TAVI management

Compared to the 2012 EuGMS TAVI survey results, more respondents reported that they know where to refer AS-patients (96% vs 75% in 2012), possibly suggesting increased availability and knowledge of TAVI pathways. AS-patients are mostly referred to specialized cardiac centres with multidisciplinary teams, and less commonly referred to general hospitals and cardiologists. The multidisciplinary 'Heart Team', including healthcare experts from various disciplines, collaboratively manages patients following a disease-specific pathway, which can span from primary to tertiary care [31]. The aim of the Heart Team is to provide a streamlined, consistent pathway which ensures that the right patients receive the right procedure at the right time [31]. It is essential for assessing surgical risk and TAVI candidacy, but it also offers a comprehensive, multiprofessional team-based approach to the diagnostic imaging assessment, preoperative planning, procedural execution, and in-hospital care for each patient undergoing this procedure [32]. Since 2012, the network linking community, district hospitals and the heart valve centres has expanded, and much work has been done to ensure that patients with heart valve disease have access to this optimal care pathway. Consistently, compared to the 2012 survey results, more respondents reported that they are part of a multidisciplinary team (38.9% vs. 20% in 2012). However, this percentage is notably modest, considering the mean age of the TAVI population, their high rate of comorbidities and prognostic relevance of geriatric assessment.

The results from the present survey indicate that geriatricians’ involvement in AS management remains scarce. Indeed, approximately the 40% of respondents reported that geriatricians rarely or very rarely participate in the pre- and post-procedural assessment of TAVI candidates. Current geriatricians’ involvement in post-procedural management is even less prevalent than it was in 2012.

To date, there is no published study on geriatric co-management for older patients undergoing TAVI. Prior initiatives aimed at improving clinical outcomes were implemented in surgical or cardiology wards, varying from preoperative comprehensive geriatric assessments to multi-component preoperative inpatient programs, prehabilitation programs, or nurse-led geriatric co-management programs within cardiology wards [33–35]. Geriatric interventions have proven beneficial for older patients in these settings, leading to improved clinically significant outcomes. Some practitioners might view preoperative comprehensive geriatric assessments as a tool to determine a patient's suitability for TAVI, but it can also serve as a foundation for geriatric co-management. There is an evident need for these interventions to undergo further evaluation in well-designed, high-quality studies. Moreover, future research should investigate the cost-effectiveness of routine geriatric assessment [36]. Recruitment challenges and limited availability of expert geriatricians might at least partly explain the poor involvement of geriatricians reported in the present survey. Our results might contribute to bringing attention to the clinical relevance of geriatric assessment in the context of AS management highlighting the need of a greater recruitment of geriatricians in public healthcare services.

### Limitations

Some limitations of the present study must be acknowledged. First, the comparability between the present (2022) and the previous (2012) survey might be limited due to some differences in the geographical distribution of participants. We are unable to determine the precise number of active EuGMS members during the dissemination period, so we cannot provide the exact number of invitations sent. Moreover, the survey was available in English only, which may have prevented participation of some non-native speaker EuGMS members. Finally, a selection bias cannot be excluded, i.e., those who were more interested in TAVI research and/or clinical management might have been more likely to participate in the survey.

## Conclusions

Geriatricians’ involvement in TAVI pathways and multidisciplinary heart teams remains scarce. More efforts should be devoted to implement geriatricians’ role in AS decision-making.

## Supplementary Information

Below is the link to the electronic supplementary material.

Supplementary file1 (DOCX 23 KB)

## Data Availability

Data is available upon reasonable request.
