# Supplementary material for: Geriatricians’ role in the management of aortic stenosis in frail older patients: a decade later
Source: Eur Geriatr Med. 2024 Jul 22;15(6):1635–43. doi: 10.1007/s41999-024-01015-9 (PMC11632017; doi:10.1007/s41999-024-01015-9)
Supplement: Supplementary file 1 — Supplementary file1 (DOCX 23 KB) [file 41999_2024_1015_MOESM1_ESM.docx]

**Supplementary Table 1.** 2012 EuGMS transcatheter aortic valve implantation (TAVI) survey.

| 1. Please indicate your gender |
| --- |
| 1. What is your medical specialty |
| 1. In which country do you currently practice medicine? |
| 1. Which of the following age categories do you fall into? |
| 1. How long have you been working in your current specialty? |
| 1. When thinking about all the patients you see and treat, approximately what share of your work is spent on acute care, long-term care and rehabilitation? |
| 1. How frequently do you manage older patients with aortic stenosis? Please give your answer on a scale of 1–7, where ‘‘1’’ means ‘‘very rarely’’ and ‘‘7’’ means ‘‘very frequently’’ |
| 1. In your experience, how frequently do the following symptoms present in older patients with aortic stenosis? Please use a scale of 1–7, where 1 means ‘‘very rarely’’ and ‘‘7’’ means ‘‘very frequently’’ |
| 1. In the past three months, approximately how many patients with aortic stenosis have you seen? |
| 1. Approximately what percentage of these patients with aortic stenosis that you saw in the past three months had mild, moderate and severe aortic stenosis,? |
| 1. In your clinical practice, how frequently are the following clinical conditions associated comorbidities in patients with aortic stenosis? Please use a scale of 1-7, where 1 means “very rarely” and 7 means “very frequently” |
| 1. Among the patients with aortic stenosis that you saw in the past three months: 2. What percentage have received medical treatment only up to this point in time, i.e. have never undergone surgery / other procedures to treat their aortic stenosis? 3. What percentage have already undergone cardiac surgery to treat their aortic stenosis? 4. What percentage have already undergone a transcatheter aortic valve implantation (TAVI) to treat their aortic stenosis? |
| 1. Please now assume that one of your patients with severe aortic valve stenosis might be suitable for transcatheter aortic valve implantation (TAVI). Would you know where to refer them? |
| 1. Where would you refer them? |
| 1. For patients with aortic stenosis who have the following conditions, which do you think would be the 3 most suitable for cardiac surgery and which would be the 3 most suitable for transcatheter aortic valve implantation (TAVI)? Please select the top 3 for each |
| 1. In the past 2 years, how many patients with aortic stenosis have you referred for a) cardiac surgery, b) transcatheter aortic valve implantation (TAVI)? |
| 1. (only if 0 for transcatheter aortic valve implantation in Q16) Why have you not referred any patients for a transcatheter aortic valve implantation (TAVI) in the past 2 years? |
| 1. You mentioned you have referred … patients (see answer from 16b) for transcatheter aortic valve implantation (TAVI) in the past 2 years. Up to now, how many of them have had:   No functional improvement ______  Functional improvement observed within 3 months of procedure ______  Functional improvement observed, but longer than 3 months after procedure ______ |
| 1. You mentioned you have referred (see answer from 16b) … patients for transcatheter aortic valve implantation (TAVI) in the past 2 years. Up to now, in how many of them have you detected an NYHA class improvement? |
| 1. You mentioned you have referred (see answer from 16b) … patients for transcatheter aortic valve implantation (TAVI) in the past 2 years. Up to now, in how many of them have you detected cognitive improvement? |
| 1. You mentioned you have referred … patients for transcatheter aortic valve implantation (TAVI) in the past 2 years. That you are aware of, what proportion of these patients suffered the following complications during the TAVI procedure? |
| 1. In your experience, how involved are the following physicians involved in the management of a patient in the lead up to a TAVI procedure? Please use a scale of 1-7, where 1 means “not involved at all” and 7 means “very involved” |
| 1. In your experience, how involved the following physicians involved the management of a patient after the TAVI procedure has been completed? Please use a scale of 1-7, where 1 means “not involved at all” and 7 means “very involved” |
| 1. Are you part of a multidisciplinary team for the management of patients who are considered for TAVI? |

**Supplementary Table 2.** 2022 EuGMS transcatheter aortic valve implantation (TAVI) survey.

| 1. Please indicate your sex |
| --- |
| 1. What is your medical specialty |
| 1. In which country do you currently practice medicine? |
| 1. Which of the following age categories do you fall into? |
| 1. How long have you been working in your current specialty? (years) |
| 1. When thinking about all the patients you see and treat, approximately what share of your work is spent on acute care, long-term care and rehabilitation? |
| 1. How frequently do you manage older patients with aortic stenosis? (Answer options: never, 1-2 times/semester, 1-2 times/month, 1-3 times/week, 3+ times/week) |
| 1. In your experience, what proportion of patients with aortic stenosis report the following symptoms? (Answer options: dyspnoea, delirium, angina, syncope, fatigue, fall during effort) |
| 1. In the past three months, what proportion of patients with aortic stenosis have you seen approximately? |
| 1. Approximately what percentage of these patients with aortic stenosis that you saw in the past three months had mild, moderate and severe aortic stenosis, respectively? |
| 1. In your clinical practice, how frequently are the following clinical conditions associated comorbidities in patients with aortic stenosis? Please use a scale of 1-5, where 1 means “very rarely”, 2 means “rarely”, 3 means “sometimes”, 4 means “often”, and 5 means “very frequently” (Answer options: coronary artery disease, hypertension, renal insufficiency, liver disease, stroke or transient ischemic attack, peripheral vascular disease, permanent atrial fibrillation, diabetes, chronic obstructive pulmonary disease, cancer, anaemia, depression, cognitive impairment, fall during effort, sarcopenia, frailty) |
| 1. Among the patients with aortic stenosis that you saw in the past three months: 2. What percentage have received medical treatment only up to this point in time, i.e., have never undergone surgery / other procedures to treat their aortic stenosis? 3. What percentage have already undergone cardiac surgery to treat their aortic stenosis? 4. What percentage have already undergone a transcatheter aortic valve implantation (TAVI) to treat their aortic stenosis? |
| 1. Please now assume that one of your patients with severe aortic valve stenosis might be suitable for transcatheter aortic valve implantation (TAVI). Would you know where to refer them? |
| 1. Where would you refer them? (Answer options: to an office-based cardiologist, to a general hospital, to a specialist heart centre / Cardiology department, to a specialist heart centre with a multidisciplinary team including a geriatrician) |
| 1. For patients with aortic stenosis who have the following conditions, which do you think would be the 3 most suitable for cardiac surgery and which would be the 3 most suitable for transcatheter aortic valve implantation (TAVI) or medical therapy? (Answer options: cancer, dementia, severe frailty, moderate-to-severe chronic kidney disease, abdominal aortic aneurysm, peripheral arterial occlusive disease, chronic obstructive pulmonary disease, recent myocardial infarction, infectious endocarditis, previous stroke or transient ischemic attack, liver failure, severe mitral insufficiency, bicuspid aortic valve, recent percutaneous coronary angioplasty) |
| 1. In the past 2 years, what proportion of patients with aortic stenosis have you referred for a) cardiac surgery, b) transcatheter aortic valve implantation (TAVI), c) medical therapy only? |
| 1. (Only if 0 for transcatheter aortic valve implantation in Q16) Why have you not referred any patients for a transcatheter aortic valve implantation (TAVI) in the past 2 years? (Answer options: patient refusal, limited life expectancy, severe frailty, other [please specify]) |
| 1. You mentioned you have referred … patients (see answer from 16b) for transcatheter aortic valve implantation (TAVI) in the past 2 years. Up to now, what proportion of them have had:   No functional improvement ______  Functional improvement observed within 3 months of procedure ______  Functional improvement observed, but longer than 3 months after procedure ______ |
| 1. You mentioned you have referred (see answer from 16b) … patients for transcatheter aortic valve implantation (TAVI) in the past 2 years. Up to now, in what proportion have you detected a NYHA class improvement? |
| 1. You mentioned you have referred (see answer from 16b) … patients for transcatheter aortic valve implantation (TAVI) in the past 2 years. Up to now, in what proportion have you detected cognitive improvement? |
| 1. You mentioned you have referred … patients for transcatheter aortic valve implantation (TAVI) in the past 2 years. That you are aware of, what proportion of these patients suffered the following complications during the TAVI procedure? Complications: stroke, major bleeding, vessel dissection, cardiac tamponade, death, other complications (please specify ____), no complications. |
| 1. In your experience, to which extent are the following physicians involved in the management of a patient in the lead up to a TAVI procedure? Please use a scale of 1-5, where 1 means “very rarely”, 2 means “rarely”, 3 means “sometimes”, 4 means “often”, and 5 means “very frequently” (Answer options: geriatrician, cardiologist, surgeon) |
| 1. In your experience, to which extent are the following physicians involved the management of a patient after the TAVI procedure has been completed? Please use a scale of 1-5, where 1 means “very rarely”, 2 means “rarely”, 3 means “sometimes”, 4 means “often”, and 5 means “very frequently”. (Answer options: geriatrician, cardiologist, surgeon) |
| 1. Are you part of a multidisciplinary team for the management of patients who are considered for TAVI procedures? |
| 1. Please mark the members of the multidisciplinary team for the management of TAVI candidates ((Answer options: geriatrician, clinical cardiologist, cardiac surgeon, patient, family, interventional cardiologist, anaesthesiologist, echocardiographer, operating room and cardiac cath lab staff, general practitioner, other physicians) |
| 1. Does patient age influence your decision to refer him/her for TAVI? If yes, please specify an age cut-off: I don´t refer for TAVI patients aged --- (i.e., >80 years / >85 years / >90 years) |
